# Do enhancements to the urban built environment improve physical activity levels among socially disadvantaged populations?

**DOI:** 10.1186/1475-9276-10-28

**Published:** 2011-07-18

**Authors:** Jamie R Pearce, Ralph Maddison

**Affiliations:** 1 Institute of Geography, School of GeoSciences, University of Edinburgh, UK; 2 Clinical Trials Research Unit, School of Population Health, University of Auckland, New Zealand

**Keywords:** Physical activity, neighbourhoods, connectivity, open space, green space, health inequalities, New Zealand

## Abstract

**Background:**

There is growing recognition that the urban built environment influences physical activity at the population level, although the effects on disadvantaged groups are less well understood. Using the examples of open/green space and street connectivity, this paper explores whether enhancements to the built environment have potential for addressing physical activity-related health inequalities among Māori, Pacific and low income communities in New Zealand.

**Method:**

A high-level review of the international literature relating open space and street connectivity to physical activity and/or related health outcomes at a population level was completed. Consideration was given to whether these features of the built environment have a disproportionate effect on disadvantaged populations.

**Results:**

Findings from international studies suggest that open space and street connectivity have a beneficial effect on physical activity. Enhancing the built environment may be particularly advantageous for improving physical activity levels among disadvantaged populations.

**Conclusion:**

It is likely that open space and street connectivity have a positive effect on physical activity behaviour; however due to the cross-sectional nature of existing research and the paucity of research among disadvantaged populations definitive conclusions about the effect in these populations cannot be made. Further research is required (e.g. natural experiments or quasi experimental research designs) to determine the effect of changing the environment on physical activity and obesity.

## Background

The prevalence of overweight among adults, children and adolescents has increased markedly in the last three decades in developed countries [1-3]. In New Zealand, over half of the adult population and almost one third of our children aged 5-14 years are overweight or obese [4,5]. Given the negative health consequences associated with being overweight [6], reducing the prevalence of overweight is justifiably a public health priority.

The high prevalence of obesity rates have been proposed to be related to various factors which promote high energy intake (eating) and sedentary behaviour, and decrease physical activity [7]. With respect to physical activity, there has been particular interest in the activity levels among socially disadvantaged populations such as ethnic minority groups, low income households, and people living in highly deprived areas. Several international reviews have generally found a positive gradient between socio-economic status (SES) and physical activity; with greater levels of leisure-time or moderate-vigorous intensity physical activity in those at the top of the socio-economic strata compared to those at the bottom [8-10]. Although lower recreational physical activity has been found in low SES neighbourhoods, racial and ethnic minorities are more likely to live in walkable neighbourhoods and walk for transportation [11]. Data from the New Zealand Children's Nutrition Survey (CNS) found that those in the highest deprivation quintile were more likely to be in the highest activity quartile compared to those in the lowest deprivation quintile. These findings mimic adult and international data and are most likely due to increased periods of active transport (such as walking to and from school). However, closer examination of the CNS data showed children in the highest deprivation quintile were significantly less likely to be active after school (when children tend to participate in structured sport and recreation activities). Caution is required when interpreting any results that uses SES indicators given the array of different economic measures used (household income versus deprivation), the age of participants, and difficulties associated with self-reported physical activity measurement.

Processes driving the low levels of physical activity are multi-faceted and operate at various levels including individual, household, community and/or societal [12]. Therefore, successful strategies for enhancing physical activity among disadvantaged populations could range from the micro- (e.g., individually targeted exercise programs) to macro-level (e.g., enhancing the built environment to encourage utilitarian and recreational physical activity) [7]. Many of the efforts to improve physical activity levels have involved interventions targeted at individuals such as advice from a general practitioner, a group seminar or a targeted physical activity program [13]. However, recent international evidence suggests that public health strategies focused on encouraging changes to individual behaviour have in isolation tended to be insufficient [7]. It is increasingly appreciated that the role of the environment is pivotal in understanding the population-level decrease in energy expenditure. Thus, a strong case can be made for substantial and sustainable environmental initiatives that provide and make easier opportunities for physical activity. Of course advocating a successful public health agenda requires a robust evidence base, including a confident assessment of whether environmental interventions do influence physical activity.

The purpose of this paper is to appraise international and New Zealand-based research to examine the impact of environmental factors on inequalities in physical activity and related health outcomes. Our particular interest is whether enhancements to the built environment have potential for addressing physical activity-related health inequalities among Māori, Pacific and low income communities in New Zealand. We focus on two key environmental factors that relate to urban design: open (green) space and street connectivity. These factors were identified as part of a larger multi-phase research study as key factors for addressing low levels of physical activity among Māori, Pacific and low income communities in New Zealand. The larger study sought to identify key intervention areas to address food security and low levels of physical activity in the three target communities. Full details of this project are detailed elsewhere [14] but in brief, we conducted literature reviews, focus groups, stakeholder workshops, and key informant interviews. Participants included members of affected communities, policy-makers, and academics. The research was informed by complexity theory [15,16] and environmental perspectives of obesity causation [17] which were used to identify key areas (control parameters) to intervene. Complexity theory recognises that social phenomena, such as nutrition and physical activity emerge from multifaceted systems with a large number of interacting elements rather than through a linear causal chain within the system. Complexity theory implies that broad changes are most likely to arise when interventions target highly linked elements of the system-the control parameters. The results of the study identified enhancing open space and connectivity as two key factors. Furthermore, green space and street connectivity are two of the most frequently researched built environmental variables. We argue that consideration of future interventions for improving physical activity should include evaluating the potential implications for inequalities in physical activity among disadvantaged groups. Whilst our focus is on New Zealand, our study will be of interest to researchers from other countries because, to our knowledge, no previous review has evaluated the evidence for whether the built environment can exert an influence on health inequalities. Of course, if there is convincing evidence to support a role for the built environment in shaping health inequalities then this assertion has important implications for policy makers tasked with addressing this important social policy concern. Health inequalities are not only unjust and ameliorable but a reduction in health inequalities has various benefits for all of society [18]. Our study provides particular attention to three socially disadvantaged population groups in New Zealand: Māori (indigenous), Pacific, and low income communities. In New Zealand, Māori, Pacific and low income communities persistently have poorer health outcomes than European populations including all-cause mortality, the leading causes of death, most types of morbidity as well as unhealthy behaviours (e.g., smoking, and poor diet) [5,19,20].

## Methods

Rather than a systematic review, we conducted a high level review or scan across the international and New Zealand literatures by searching pertinent reviews (systematic and non-systematic) that have been completed on the built environment and their implications for physical activity levels. In addition, papers were sought using key search engines including Cinahl, CDSR, Central/CCTR, PubMed, PsychInfo, SportsDiscus, and Google Scholar to identify articles that were either published after or beyond the scope of the published reviews and yet added further insights. The search engines were used to identify research pertaining to open space and connectivity. Search terms included 'open space', 'green space', 'connectivity', 'physical activity', 'walking', 'exercise', 'obesity', 'BMI', 'neighbourhoods', 'parks', 'environmental', 'greenness', and 'built environment' This evaluation of existing research was limited to reviews published in English before January 2009. Reviews had to relate to environmental determinants of physical activity. Reviews that were related to obesity or obesogenic environments but did not clearly differentiate environmental determinants related to physical activity were not included. The evidence base for New Zealand studies was extended to include reviews and individual studies.

Each of the papers included in the published reviews and those found using the search were examined to firstly assess the strength of the evidence linking connectivity and open space to physical activity and/or related health outcomes. Second we evaluated whether there was evidence to suggest that these features of the built environment had a disproportionate effect on disadvantaged populations (and hence impact on inequalities in physical activity or related health outcomes). We anticipate that these findings will be of concern to researchers with interests in the role of the environment in understanding health inequalities particularly with regards to physical activity. Further, the results have salience for policy makers in New Zealand and elsewhere who are tasked with developing novel interventions for improving physical activity levels among disadvantaged communities.

## Results

Results are presented in two stages. First, the findings from the international literature scan are provided before moving to the New Zealand-specific studies in the second part of the results section. The two sets of results are further stratified into research pertaining to open space and then street connectivity.

### A. International review

#### Improving urban design: open space

There is a growing literature identifying a link between neighbourhood open space and the physical activity levels of local residents. Open space refers to a variety of definitions including designated urban parks which often include recreational facilities such as play areas for children, as well as broader designations that encompass some or all forms of green space. Locational access to open and green space provides the opportunity structures within the neighbourhood for walking, cycling and other forms of physical activity. This body of work has been the subject of a number of international reviews [21-25], with much of the research undertaken in the United States (U.S.). In general, the international evidence is supportive of an effect of open/green space on levels of physical activity and related health outcomes. Some studies have utilised direct (objective) measures of locational access to open space (Geographical Information Systems [GIS] measures) and modelled the effects on the physical activity levels of local residents. Access was captured in a variety of ways including: the proportion of green space in a predefined geographical unit that approximates a neighbourhood (using GIS layers from land use databases or remotely sensed images); 'mental maps' of local environments (i.e. personalised view of the neighbourhood); count of open spaces within a set distance of each household/neighbourhood centroid; distance through the road network from place of residence to closest green space; and environmental audits using methods such as systematic social observation. Rather than using objective measures of the local environment, other studies have relied on people's perceptions of their recreational environment [22]. The research also covers a variety of outcome measures including total physical activity, vigorous intensity activity, and the amount of time spent walking for exercise or for transport [22,24]. Studies in the U.S. have found that the density of recreational resources (including open space) are associated with the physical activity levels among both adults [26-28], older adults [29,30], and children [21]. In some instances, the quality of, and facilities available in, open spaces such as parks were found to be more important than simple locational access [31]. Similar findings have been noted in other countries, with a particularly strong evidence base in Australia [32-39]. Other types of open space, such as access to beaches have also been shown to be associated with increased physical activity levels [40]. Neighbourhood greenness (or sometimes defined as 'natural environments') has been associated with higher levels of physical activity and/or lower BMI scores among adults [41-46] and children [47]. In addition, there is compelling evidence that green space is associated with broader measures of health such as perceived general health [41,46,48,49], an effect that is potentially mediated by enhanced physical activity levels.

In terms of health inequalities, there is mounting evidence from various countries that more socially deprived communities have poorer locational access to open/green space [50-52], although this finding is not observed in all settings [53-55]. Research has also found that the quality of the open space (e.g., well lit, high grade facilities etc) varies between areas stratified by area-level deprivation with less disadvantaged areas tending to have better quality space [56,57]. The observation that the environmental characteristics (including those that promote physical activity) in more socially deprived areas are systematically inferior to those in higher income areas has been termed 'deprivation amplification' [58,59]. However, closer scrutiny of the distribution of resources across deprived and non-deprived neighbourhoods has led some researchers to question this assumption [55,60]. Fewer studies have directly considered (using health outcome data) the implications of differential access to open space on health inequalities. One U.S. observational study in a selection of ethnic minority and low income neighbourhoods found that, unlike the general population, public parks were the most common sites for exercise [28]. In turn, the utilisation of parks and physical activity levels were strongly influenced by the distance between participant's home and the parks included in the study. These findings suggest that access to open space may influence physical activity levels to a greater extent for disadvantaged communities. Similarly, a study in Australia demonstrated that access to open space mediated women's educational inequalities (a proxy for socioeconomic status) in leisure time walking (but not walking for transport) [36]. These latter findings were supported by a study of women in the U.S. [61]. Together, these results suggest that women who are on a low income or living in low SES neighbourhoods may disproportionately benefit from greater availability of physical activity resources. A study in small areas across England found that, after adjustment for potential confounders, level of greenness moderated the relationship between area-level deprivation for various health outcomes that are plausibly linked to physical activity [62]. The findings are significant because they suggest that good locational access to green space may attenuate health inequalities.

In summary, whilst there is a paucity of literature evaluating the influence of access to open space on disadvantaged groups, the evidence that is available suggests that better locational access in more disadvantaged areas is particularly beneficial to these groups and the implementation of suitably designed initiatives has potential to reduce inequalities in physical activity (and related health outcomes). However, whilst most studies have found neighbourhood open space to be associated with increased physical activity levels, the size of this effect suggests that improving access alone is unlikely to be sufficient in increasing physical activity levels to recommended levels [35]. Therefore, interventions to improve access to open space require complementary strategies which aim to influence individual and socio-environmental factors.

#### Improving urban design: street connectivity

There is a small but growing body of research examining the effects of street connectivity on physical activity and related health outcomes. Whilst precise operational definitions of connectivity vary, neighbourhoods with a high degree of connectivity are designed with a well-connected street network with plentiful intersections, small block sizes and few cul-de-sacs. High levels of connectivity can facilitate walking through active transport and increase overall physical activity by providing shorter trips to a larger range of destinations within easy reach, reducing the speed of traffic, and decreasing reliance on private forms of transport [63,64].

Most research into the influence of street connectivity on individual-level physical activity comes from the U.S. This body of research has overwhelmingly demonstrated that greater levels of connectivity are related with higher levels of physical activity and lower prevalence of physical activity-related morbidity and mortality measures. U.S. studies have generally found small but statistically significant associations between street connectivity and total and/or vigorous physical activity [29,65-68]. For example, using data collected from children wearing accelerometers and GIS measures of neighbourhood connectivity it was found that street connectivity accounted for an additional six percent (after adjustment for sex) of the variance in objectively measured physical activity [67]. Similar findings were found among older women [69]. Some studies have found the effects of connectivity on physical activity to be limited to leisure activity rather than utilitarian forms [70]. Others have found the effects to be restricted to younger people [71]. However, not all U.S. studies have noted a significant association between connectivity and physical activity [72]. For instance, a study in Atlanta found that whilst other characteristics of the built environment (e.g. land use mix) were significant, local street connectivity was not associated with overall physical activity levels of local residents [73].

Outside of the U.S. there is a paucity of research into street connectivity and physical activity. One study in an urban area in Queensland, Australia using GIS-derived measures of street connectivity to local parkland found counterintuitive associations with self-reported physical activity [74]. The results suggested that respondents who had unacceptable connectivity to parkland were more likely to attain sufficient levels of physical activity than those who had more direct locational access. In a sample of 705 adolescent girls (mean age 14.7 years) in the Aveiro District of Portugal, street connectivity was a predictor of active transportation [75].

Other research has considered physical activity-related health outcomes such as obesity. Studies examining the effect of neighbourhood connectivity (objectively measured using GIS) on body weight are scarce and many [73,76-78], although not all [79-81], have found there to be an association. However, even when a positive association was noted the effect was not consistent across all social and demographic groups and/or all measures of weight/obesity.

Few studies have explicitly compared the effects of connectivity on different social groups, and considered the implications for health inequalities. In a sample of 1282 Australian women it was found that street connectivity in each participant's neighbourhood mediated inequalities (measured between educational groups: a proxy for socio-economic status) in physical activity for transport but not for leisure [36]. The authors suggested that public health strategies to reduce social inequalities in physical activity could usefully focus on environmental strategies such as modifications to the built environment. However, a study in the southern U.S. found that the positive effects of street connectivity on physical activity levels and BMI were restricted to non-Hispanic White populations, and there was no significant effects for African Americans [82].

In summary, the review of the international literature suggests that neighbourhood connectivity has a modest but important role to play in influencing physical activity and related health outcomes. However, the total number of studies is low with very few completed outside of the U.S. Unsurprisingly, few researchers have considered the implications of street connectivity for inequalities in physical activity and related health outcomes. This area is clearly a key field for future investigation.

### B. National review

#### Improving urban design: open space

Whilst the evidence base around open space, connectivity, and physical activity is well developed in countries such as the U.S. and Australia, in New Zealand the field of research is in its infancy. Recent research used a GIS approach to develop a national index of access to a variety of health-related 'community resources' for neighbourhoods across the country [83]. Among the community resources of interest were access to open space and beaches, which were measured using the travel time from the centroid of each neighbourhood (n = 38,254) to the closest park and beach. Analysis of the index found that locational access to open space (parks) was better in more deprived neighbourhoods (measured using the New Zealand Deprivation Index) across New Zealand, suggesting a pro-equity distribution [60]. For beach access, there was no relationship with area deprivation. However, these national-level trends were not consistent in rural areas and in some regions of the country with high proportions of Māori and low income populations, where the opposite pattern could be observed [84].

With regards to any association between access to open space and physical activity related health outcomes, the research team used the New Zealand Health Survey [5] (12,529 participants in 1178 neighbourhoods across the country) and appended the neighbourhood measures of access. They found that neighbourhood access to parks was not associated with BMI, sedentary behaviour or physical activity, after controlling for individual-level socio-economic variables, and neighbourhood-level deprivation and urban/rural status. There was some evidence of a relationship between beach access and BMI and physical activity in the expected direction [85]. Although this is the only local study and uses only a single measure of open space, it suggests that there is little evidence of an association between locational access to open spaces and physical activity in New Zealand. The authors speculate that the discrepancy between the international findings and those in New Zealand may be because of the lack of variation in the exposure in neighbourhood exposure variables. In other words, most neighbourhoods in New Zealand have relatively good access to open space.

#### Street connectivity

Studies of physical activity and street connectivity in New Zealand are equally scarce. One study was undertaken in Auckland (the largest city) and used a measure of the node ratio (derived by dividing the number of street intersection nodes by the number of intersection and cul-de-sac nodes contained within a 500 m buffer zone of a respondent's commute route) to investigate transport-related physical activity behaviour [86]. Using logistic regression, the authors found evidence that street connectivity was related to transport-related physical activity. Respondents who commuted through the most connected streets were more likely to engage in transport-related physical activity modes to access their occupation (Odds Ratio = 6.9) when compared to those traveling along the least connected. None of the other urban design variables that the authors used were found to be related to physical activity.

Most recently, a study was conducted in Auckland, New Zealand to determine the feasibility of integrating environmental, individual-level, and psychosocial variables to better understand adolescents' physical activity [87]. Although small (n = 110) this study included objective (GIS) (walkability and accessibility) and perceived measures (access to physical activity facilities) of the environment. Using structural equation modeling, results showed that walkability and accessibility were not related to physical activity, measured both with accelerometers and self-reports.

In summary, the New Zealand evidence base for the effect of the built environment on health is extremely limited. There has been no research that has explicitly examined the effects of the built environment on the three target populations. This omission is perhaps unsurprising given that even in the U.S. where this literature is relatively well developed, there have only been 10 studies to examine the effects of the built environment (all components, not just open space and connectivity) on the largest ethnic minority group in that country (African-American) [88]. The New Zealand studies that have evaluated associations between connectivity, open space and physical activity are not generally consistent with the international literature and are not supportive of an effect. Nonetheless, it would be unwise to dismiss these urban design variables as being unimportant in the New Zealand context. Because of the small number of studies (n = 3), as well as the methodological limitations and the data constraints of previous work, the evidence base is not sufficiently developed for definitive conclusions.

## Discussion

There is growing international evidence that various physical features of the built environment are pertinent in understanding individual-level physical activity levels. Neighbourhood characteristics that potentially influence physical activity levels include: access to recreational facilities; neighbourhood aesthetics; social capital; crime and incivilities; and transportation options. This paper has focused on two key physical activity-related characteristics of the built environment: open space and street connectivity. A review of the potential implications of these features of the built environment for inequalities in physical activity has been provided. In particular, consideration has been given to the potential to improve physical activity levels among three disadvantaged groups in New Zealand (Māori, Pacific and low income communities).

The international research into the effects of open space and connectivity on physical activity and related health outcomes is promising. Residents living in neighbourhoods that are characterised by having good locational access to green space and which have networks of streets, paths and cycleways that are highly connected are often more physically active. Most research on the influence of the built environment (including open space and connectivity) has been undertaken in the U.S. This work is generally supportive of a small positive and statistically significant effect. However, due to the difficulty of capturing detailed longitudinal data, the vast majority of studies are cross-sectional or, at best, use a repeated cross-sectional design. This study design is a major impediment to developing successful urban design policy initiatives to combat low levels of physical activity. It is not possible to determine causality from cross-sectional analyses due to the possible effects of reverse causality (or 'endogeneity'/'self selection'). Reverse causality could occur, for example, when residents who are physically active preferentially locate in areas that are conducive to physical activity. Further, the evidence base in New Zealand is extremely limited with only three studies completed. Results have generally not found a significant relationship between the built environment and physical activity.

There is a dearth of international research examining the influence of the built environment on the levels of physical activity (and related health outcomes) among disadvantaged populations. Existing research in the U.S. has focused on low income and/or African-American populations. In New Zealand, there has been no research evaluating the effect of any component of the built environment (including open space and connectivity) on the three target populations. Whilst the spatial targeting of policy initiatives may address physical activity levels of residents in those localities, it is important to note that Māori, Pacific and low-income peoples in New Zealand are not heavily segregated [89,90].

Adapting the built environment (including open space and connectivity) is potentially a long term and sustainable approach to addressing physical activity levels at the population level. Interventions to alter aspects of the built environment are likely to prove far reaching and durable. They are likely to support positive changes to behaviour, practices and attitudes to physical activity. Despite this, there is very little research that has examined the direct effect of changing the built environment on physical activity. Research is clearly needed in this area and utilising natural experiments or quasi experimental research designs would be useful approaches. For instance, well-designed evaluations of interventions such as urban regeneration projects in high social deprivation localities that seek to improve the health and well-being (including physical activity-related outcomes) of local residents is a potentially fruitful approach for understanding the influence of the built environment on disadvantaged and low income communities. Addressing issues relating to the built environment will require collaboration across a range of sectors including those working in planning/urban design at the national and local level, architects, sports and recreation, transportation as well as the public health community. Changing existing environments is not without potential side-effects. For example, possible side-effects include changes to traffic flows in major urban areas and the associated costs for businesses and households. More positive side effects may also be envisaged such as the development of more sustainable cities, higher levels of social capital and social cohesion, as well as a reduction in car dependency and the associated lowering of pollution levels.

Comparing findings across studies is also problematic. It is important to consider the sensitivity of the research findings that are outlined above to the methods used to capture the built environment. Objective (often GIS-based) measures have been applied in multiple ways (e.g. at various geographical scales and/or capturing subtly different aspects of the environment) which limits the comparability of the findings across settings. Further, the ways in which the effects of the built environment are mediated by local residents' perceptions of the environment and their physical activity levels are not well understood. Similarly, a better understanding of the role of the built environment in shaping inequalities in physical activity can be realised through more nuanced investigations that consider a variety of physical activity outcomes and socio-demographic markers. For instance further work that scrutinises the influence of the built environment on utilitarian (for transport) and recreational physical activity is warranted and offers considerable policy insights. Further, whether the built environment disproportionately affects inequalities in physical activity at specific points in the life course (e.g. children or older age groups) or has different effects on males and females is also poorly understood. Similar arguments can be formulated for different ethnic groups.

A policy approach to changing the environment could be the implementation of a National Policy Statement (NPS). NPSs enable the national (New Zealand) government to prescribe objectives and policies on resource management matters of national significance that are pertinent to promoting the sustainable management of natural and physical resources. NPSs steer subsequent decision-making at the national, regional and district levels and hence can significantly influence resource management practices in New Zealand. Historically, city zoning ordinances have tended to be implemented with a view to separating residential neighbourhoods from industrial facilities to limit residents from the harmful effects (particularly health) of exposure to negative externalities such as infectious disease and industrial air pollution. However, with the shift in focus over the course of the twentieth century from infectious to chronic diseases such as obesity, public health concerns have largely become absent from major planning or land use decision making. With regards to improving physical activity among minority of low socioeconomic populations, a NPS may have the potential to provide the framework for the implementation of any urban design recommendations (e.g. enhancing green space and/or connectivity). A NPS on urban design 'can be a potent tool in creating a built environment that is conducive to public health' (p9) [91]. Various avenues are possible within a NPS framework including zoning, enhancing greenspace and ensuring that neighbourhoods have high degrees of connectivity.

## Conclusion

In conclusion, it is likely that open space and street connectivity have a positive effect on physical activity behaviour, which would be related to beneficial outcomes; however due to the cross-sectional nature of existing research and the paucity of research among disadvantaged populations, definitive conclusions about the effect on these populations cannot be made. Further research is clearly needed in this area to determine the effect on physical activity and of changing the environment. It seems that the potential benefits associated with having an environment that promotes physical activity (more sustainable cities, higher levels of social capital and social cohesion, reduction in car dependency and the associated lowering of pollution levels, as well as improvements in health) outweigh the potential side effects (changes in traffic flows and the associated costs for businesses). From a solution-oriented perspective [92], environmental changes that promote physical activity and prevent obesity are required now. Natural experiments and similarly designed research projects are needed to evaluate the effect of these environmental changes, rather than persevering with cross-sectional research to identify associations.

## Competing interests

The authors declare that they have no competing interests.

## Authors' contributions

JP led the scan of the literature and completed the first draft of the manuscript. RM participated in the design of the study and helped to draft the manuscript. Both authors read and approved the final manuscript.
